# Supplementary material for: Evaluation of circulating tumor cells as a prognostic biomarker for early recurrence in stage II–III breast cancer patients using CytoSorter® system: a retrospective study
Source: PeerJ. 2021 Apr 29;9:e11366. doi: 10.7717/peerj.11366 (PMC8088762; doi:10.7717/peerj.11366)
Supplement: Supplemental Information 2 [file peerj-09-11366-s002.docx]

**Table S1.** Correlation of CTCs with patient demographics and clinicopathological characteristics.

| Parameter | n | Average CTC Count ( per 4 mL) | *P* |
| --- | --- | --- | --- |
| **Age** |  |  |  |
| < 53 | 19 | 2.53 | 0.8466 |
| ≧ 53 | 17 | 2.59 |  |
| **TNM stage** |  |  |  |
| II | 29 | 2.21 | 0.0462 |
| III | 7 | 3.75 |  |
| **T stage** |  |  |  |
| T1 | 5 | 2.00 | 0.027 |
| T2 | 28 | 2.32 |  |
| T3 | 3 | 5.67 |  |
| **N stage** |  |  |  |
| N0 | 15 | 2.07 | 0.3251 |
| N1 | 14 | 2.71 |  |
| N2 | 5 | 2.40 |  |
| N3 | 2 | 5.50 |  |
| **Differentiation** |  |  |  |
| I | 13 | 2.93 | 0.1363 |
| II | 11 | 2.91 |  |
| III | 12 | 1.73 |  |
| **ER** |  |  |  |
| Yes | 27 | 2.37 | 0.5154 |
| No | 9 | 3.11 |  |
| **PR** |  |  |  |
| Yes | 21 | 2.19 | 0.203 |
| No | 15 | 3.07 |  |
| **HER2** |  |  |  |
| Yes | 10 | 2.80 | 0.4188 |
| No | 26 | 2.46 |  |
| **Menopause** |  |  |  |
| Yes | 21 | 2.95 | 0.1861 |
| No | 15 | 2.00 |  |
| **Relapse** |  |  |  |
| Yes | 9 | 3.78 | 0.0224 |
| No | 27 | 2.15 |  |

Abbreviation: n = number; CTCs = circulating tumor cells; TNM = tumor-node-metastasis; ER = estrogen receptor; PR = progesterone receptor; HER2 = human epidermal growth factor receptor-2.
