# Supplementary material for: Evaluation of circulating tumor cells as a prognostic biomarker for early recurrence in stage II–III breast cancer patients using CytoSorter® system: a retrospective study
Source: PeerJ. 2021 Apr 29;9:e11366. doi: 10.7717/peerj.11366 (PMC8088762; doi:10.7717/peerj.11366)
Supplement: Supplemental Information 3 [file peerj-09-11366-s003.docx]

**Table S2.** Correlation of recurrence and recurrence-free-survival (RFS) with CTCs with different cut-off values.

| Parameter | n | Recurrence | *P* | *RFS (P)** | D-recurrence | *P* | *D-RFS (P)** |
| --- | --- | --- | --- | --- | --- | --- | --- |
| **CTCs** | |  |  |  |  |  |  |
| ≧ 1 | 32 | 9 | 0.5531 | 0.233 | 7 | 0.5658 | 0.292 |
| < 1 | 4 | 0 |  |  | 0 |  |  |
| ≧ 2 | 25 | 8 | 0.2225 | 0.148 | 6 | 0.3999 | 0.29 |
| < 2 | 11 | 1 |  |  | 1 |  |  |
| ≧ 3 | 18 | 7 | 0.1212 | 0.055 | 5 | 0.4018 | 0.191 |
| < 3 | 18 | 2 |  |  | 2 |  |  |
| ≧ 4 | 9 | 5 | 0.0262 | 0.013 | 4 | 0.0497 | 0.029 |
| < 4 | 27 | 4 |  |  | 3 |  |  |

Abbreviation: n = number; RFS = recurrence-free survival; D =distant; D-RFS =distant recurrence-free survival; CTCs = circulating tumor cells.

* Follow-up (days): min = 128; max = 723; mean = 518; median = 536.
